# Quantifying the under-estimation of cervical Cancer in remote regions of Tanzania

**DOI:** 10.1186/s12885-020-07439-3

**Published:** 2020-09-30

**Authors:** Mariah P. Gesink, Robert M. Chamberlain, Julius Mwaiselage, Crispin Kahesa, Kahima Jackson, William Mueller, Jane L. Meza, Amr S. Soliman

**Affiliations:** 1 College of Public Health, University of Nebraska Medical School, Omaha, NE USA; 2 City University of New York Medical School, 160 Convent Avenue, New York, NY 10031 USA; 3 The University of Texas M. D. Anderson Cancer Center, Houston, TX USA; 4 Ocean Road Cancer Institute, Dar es Salaam, Tanzania; 5 Bugando Hospital, Mwanza, Tanzania; 6 Mbeya Referral Hospital, Mbeya, Tanzania

**Keywords:** Cervical cancer, Incidence, Tanzania, Observed, Expected, Epidemiology

## Abstract

**Background:**

Cervical cancer is the most common cancer among women in Sub-Saharan countries, including Tanzania. While early detection and diagnosis are available in some parts of this large country, radiotherapy has been only available at the Ocean Road Cancer Institute (ORCI), in the capital city of Dar es Salaam and is just starting in a few regions.

**Methods:**

The objective of this study was to compare the observed incidence of cervical cancer for the two remote regions of Mwanza in western Tanzania and Mbeya in southern Tanzania, based on their patients treated at the ORCI from 2011 to 2014. **Results:** The number patients referred and treated at ORCI were (120 from Mwanza, and 171 from Mbeya, representing 24.6 and 32.8% of the patients histopathologically confirmed in the two sites, respectively. The results showed significant underestimation of cervical cancer in the two regions. The vast majority of patients who were histopathologically-confirmed in their local regions (73.92% from Mwanza and 65.1% from Mbeya), but did not receive the needed radiotherapy treatment at the ORCI. The estimated incidence for the two regions based on the number of patients treated at the ORCI were underestimated by 53.9% for Mwanza and 68.9% for Mbeya.

**Conclusions:**

Local establishment of radiotherapy treatment facilities in remote regions in Tanzania and similar other low-income countries is essential for providing effective treatment and improving survival of diagnosed cervical cancer patients. Linkage between the records of local remote hospitals and the main cancer treatment center in the capital city can also help support the emerging the population-based cancer registry at ORCI.

## Background

Globally, about 569,847new cervical cancer cases were reported and over a quarter of a million (311,365 deaths) occurred in 2018 [1]. The vast majority of the global burden of cervical cancer (85% of cases) occurs in developing countries [2]. Cervical cancer is the most common cancer among women in Tanzania with about 9772 new cases each year and incidence rates of 59.1 per 100,000 compared to a global disease incidence of 13.1 per 100,000 [3]. About 6695 women in Tanzania die each year from cervical cancer because of the advanced stages at diagnosis and lack of effective treatment [4]. Many studies have shown the need for effective treatment of cervical cancer unless the disease is diagnosed at early stages [5, 6] or prevented by HPV vaccination [7].

The Ocean Road Cancer Institute (ORCI) in Dar es Salaam, Tanzania, is the only specialized cancer treatment center in Tanzania that provides radiotherapy. Women with cervical cancer who reside in remote regions of the country must travel a long distances and arrange accommodation in Dar es Salaam to receive the necessary radiotherapy treatment for advanced cancer at the ORCI [6, 8]. Women can receive cervical cancer screening at outreach sites that are located in the majority of the regions of Tanzania, but these are not well utilized by women because of limited awareness [9, 10] and limited number of clinics or screening days within maternal health services of the clinics.

Because screening and treatment are limited and due to the absence of a population-based cancer registry in Tanzania, the incidence of the cervical cancer is believed to be inaccurate and possibly underestimated. Therefore, we conducted this study to quantify the incidence of cervical cancer for the two remote regions of Mwanza and Mbeya.

## Methods

### Study sites

Tanzania is a country of 885,800 km^2^ geographic area and estimated population of 58,005,463 in 2019. The country is divided into 30 regions, and Dar es Salaam region has the largest population of approximately 6,368,000 individuals. Mwanza region, on the shore of Lake Victoria, has the second largest population of approximately 2,772,509 individuals with five district hospitals and a referral hospital that offers diagnostic pathology and chemotherapy for cancer patients. Mbeya region, in the southern part of Tanzania, has the third largest population of approximately 2,707,410 individuals and has three district hospitals and a referral hospital that offers diagnostic pathology and chemotherapy for cancer patients.

### Data sources

In the region of Dar es Salaam, data were abstracted from medical records of the ORCI, the only cancer center in Tanzania that offers both chemotherapy and radiotherapy at the time of conducting the study. In the region of Mwanza, data were obtained from the Bugando referral hospital. In the region of Mbeya, data were obtained from the Mbeya referral hospital.

This retrospective study took place between April 2016 and March 2018. Data were obtained from admission logbooks, patient medical records, and pathology reports for the period of 2011–2014 from the three study hospitals.

Tanzania utilizes a referral system that starts at the village level and works up to district, regional, and finally referral hospitals in the different regions then to the capital city, if needed. There are four referral hospitals in the country, the Muhimbili National Hospital (MNH) which serves the eastern zone; Kilimanjaro Christian Medical Center (KCMC) which serves the northern zone; Bugando referral hospital which serves the western zone; and the Mbeya referral hospital which serves the southern highlands. Bugando and Mbeya referral hospitals both have pathology laboratories and offer chemotherapy. Radiotherapy started at Bugando in 2019. The ORCI is a specialty cancer hospital and the only cancer center in Tanzania that provides both chemo- and radiotherapy for cancer treatment.

### Data collection

#### The ORCI

The ORCI logbooks were first used to identify cervical cancer patients. Individual logbooks of each study year (2011–2014) contained patient name, age, sex, medical record number, place/region of residence, and date and place of confirmed cervical cancer diagnosis, and date of admission/treatment at the ORCI. All medical records were retrieved, and cervical cancer diagnosis confirmed from the medical records and the following additional information was abstracted for each patient: HIV status, referral hospital, histopathologic diagnosis, stage at diagnosis, and treatment plan. The very limited number of patients who are diagnosed at FIGO 1b are treated in the surgery department of the Muhimbili National Hospital and do not come to ORCI, as mentioned in our previous publication [8]. Only FIGO II and higher are eligible for radiotherapy.

#### Bugando and Mbeya hospitals

At each of the Bugando and Mwanza hospitals, histopathologic reports of all cervical biopsy lesions examined during the study period of 2011–2014, were retrieved and abstracted regarding the following variables: patient name, age, hospital/pathology ID number, place or residence, and details and date of diagnosis.

#### Data management and analysis

Age, year of diagnosis, and region/place of residence were used together to identify duplicates between ORCI and the two referral hospitals. The Tanzania National Bureau of Statistics 2012 Census of Population and Housing report data were used for obtaining the population denominators for 2011 and 2012. For the years of 2013 and 2014, geometric population projection method was applied based on the 2002–2012 regional annual average population growth rates for the population denominators. The denominator used in all calculations was the adult female population. The Tanzania HIV/AIDS and Malaria Indicator Survey (THMIS) 2012 reported the HIV prevalence in the female reproductive age group of 15 to 49 years as 8.2% in Dar es Salaam, 4.7% in Mwanza, and 11.0% in Mbeya. Direct proportion adjustment was applied to calculate expected incidence rates in the regions of Mwanza and Mbeya based on the observed Dar es Salaam region incidence, adjusting for respective regional HIV prevalence.

The observed (inferred) incidence rates for Mwanza and Mbeya were calculated using data of the patients from the two regions who were treated at ORCI only. The expected incidence rates for Mwanza and Mbeya were calculated based on the observed incidence rate for the region of Dar es Salaam. We assumed that both Mwanza and Mbeya had similar risk factors to Dar es Salaam, but we adjusted for the local HIV prevalence of Mwanza and Mbeya.

After reviewing the data from ORCI and the referral hospitals, we calculated the observed and expected incidence for Mwanza and Mbeya. The observed incidence was calculated by dividing the number of cervical cancer cases from each of Mwanza and Mbeya that were treated at ORCI by the estimated population denominator for each region, as described above. The expected incidence for each region was calculated by projecting the incidence for each of Mwanza and Mbeya based on the actual incidence of cervical cancer of Dar es Salaam to the population of each of Mwanza and Mbeya and considering the HIV rate in each site“. In other words, what would be the expected incidence in Mwanza and Mbeya if they had the incidence rate of Dar es Salaam and considering their different population denominator and HIV rates? Dar es Salaam was used as the reference site to project from for the other two sites because Dar es Salaam has the least possible missed cases due to accessibility to care at ORCI.

Two-independent sample *t* tests were used to compare the mean values between groups, and the chi-square test was used for comparison of proportions between groups. Kruskal-Wallis non-parametric test was used for comparing the observed and expected data points between observed and expected incidence for each of the 2 sites. A *p*-value of < 0.05 was considered significant.

The study was approved by the institutional review boards for the protection of human subjects of the University of Nebraska Medical Center and the Ocean Road Cancer Institute in Tanzania.

## Results

At the Bugando hospital in the Mwanza region, of the 1560 identified cervical biopsies in 2011–2014, 657 (42%) were histopathologically confirmed cervical cancers, and 903 (58%) were non-cancers or missing. At the Mbeya hospital, of the 1030 identified cervical biopsies, 798 (77%) were histopathologically confirmed cervical cancers, and 232 (23%) were non-cancers or missing. Non-cancers included cervical intraepithelial neoplasia (*n* = 44, 23.8%), cervicitis (*n* = 26, 14.1%), low-grade squamous intraepithelial lesions (*n =* 2, 0.01%), other infections (*n* = 40, 21.6%), normal (*n* = 29, 15.7%), and missing (*n =* 44, 23.8%).

Table 1, shows the distribution of cervical cancer cases by place of identification. Both Mwanza and Mbeya had the majority of their cervical cancer patients treated at the local respective hospitals, meaning that the patients never made it to ORCI for treatment. But there was a significant difference between Mwanza and Mbeya regarding where the cases were identified (*P* < 0.001).

**Table 1:** Distribution of Cervical Cancer Patients from Mwanza and Mbeya Who Were Managed at Ocean Road Cancer Institute (ORCI) or Diagnosed Locally but Not Treated at ORCI

	ORCI	Regional hospital then ORCI	Regional hospital only^*^	Total
	No. (%)	No. (%)	No. (%)	
Mwanza				
2014	17 (10.18)	27 (16.17)	123 (73.65)	167
2013	16 (8.74)	20 (10.93)	147 (80.33)	183
2012	6 (3.61)	39 (23.49)	121 (72.89)	166
2011	10 (7.09	34 (24.11)	97 (68.79)	141
Mbeya				
2014	25 (11.36)	41 (18.64)	154 (70.00)	220
2013	25 (12.38)	42 (20.79)	135 (66.83)	202
2012	30 (15.46)	51 (26.20)	113 (58.25)	194
2011	26 (14.29)	37 (20.33)	119 (65.38)	182

^*^Comparison of the patients who were treated at the regional hospital versus those who were treated at OCI and the *P* values are included in Table 4

For the years 2011 to 2014, Mwanza had an average of 7.41% of cervical cancer cases found only at ORCI. Also, an average of 18.68% of cervical cancer cases found both at Bugando Hospital and ORCI and an average of 73.92% of cervical cancer cases found only at Bugando hospital. For the years 2011 to 2014, Mbeya had an average of 13.37% of the cervical cancer cases found only at ORCI, an average of 21.51% of the cervical cancer cases found both at Mbeya hospital and ORCI, and an average of 65.12% of the cervical cancer cases found only at the Mbeya hospital.

Table 2, illustrates the demographic characteristics of patients seen at ORCI. The mean (SD) age at diagnosis was 50.6 (12.42) and 52.1 (11.41) years for Mwanza and Mbeya regions, respectively. Regarding HIV, 81.07% of women from Mwanza and 80.14% of women from Mbeya had missing HIV status. There was no significant difference between age of patients from the two regions (*P* = 0.17), year of diagnosis (*P* = 0.87), marital status (*P* = 0.32), or HIV status (*P* = 0.97).

**Table 2:** Demographic Characteristics of Cervical Cancer Patients Managed at ORCI 2011–2014 from Mwanza and Mbeya

	Mwanza (169)	Mbeya (277)	P^*^
Age			
Mean ± SD	50.56 ± 12.42	52.15 ± 11.41	0.17
Median	50	51	
Range	62 (18–80)	54 (26–80)	
Year of Diagnosis			
2014	44 (26.04)	63 (22.74)	0.87
2013	45 (26.63)	81 (29.24)	
2012	36 (21.30)	67 (24.19)	
2011	44 (26.04)	66 (23.83)	
Marital Status			
Single	11 (6.51)	10 (3.61)	0.32
Married	84 (49.70)	137 (49.46)	
Divorced	21 (12.43)	16 (5.78)	
Widowed	21 (12.43)	72 (25.99)	
Unknown	32 (18.93)	42 (15.16)	
HIV status			
Negative	16 (9.47)	23 (8.30)	0.97
Positive	16 (9.47)	32 (11.55)	
Unknown	137 (81.07)	222 (80.14)	

^*^t-test was used for the age comparison and chi-square tests for the remainder of the variables in the table

Table 3, shows the clinical characteristics of patients seen at ORCI. Again, there were no significant differences between the two regions with respect to cancer stage (*P* = 0.96), course of treatment (*P* = 0.21), or histopathologic type of lesions (*P* = 0.68). For patients whose histopathology was known, most of them had squamous cell carcinoma, 78.11% in Mwanza and 81.59% in Mbeya. For patients whose cancer stage was noted, most were diagnosed with stage II, 45.56% in Mwanza and 53.79% in Mbeya. Usual treatment protocols indicate that patients with Stages II and above should receive radiotherapy.

**Table 3:** Clinical Characteristics of Cervical Cancer Patients Managed at ORCI 2011–2014 from Mwanza and Mbeya

	Mwanza (169)	Mbeya (277)	P^*^
	No. (%)	No (%)	
Stage			
I	10 (5.92)	11 (3.97)	0.96
II	77 (45.56)	149 (53.79)	
III	52 (30.77)	78 (28.16)	
IV	13 (7.69)	17 (6.14)	
Unknown	17 (10.06)	22 (7.94)	
Treatment			
Radiotherapy	64 (37.87)	110 (39.71)	0.21
Radio &Chemotherapy	72 (42.60)	130 (46.93)	
Palliative	6 (3.55)	8 (2.89)	
Unknown	26 (15.38)	29 (10.47)	
Refused treatment	1 (0.59)	0 (0.00)	
Hisopathologic type			
Squamous cell carcinoma	132 (78.11)	226 (81.59)	0.68
Adenocarcinoma	14 (8.28)	21 (7.58)	
Carcinoma (non-specific)	2 (1.18)	3 (1.08)	
Clear cell carcinoma	4 (2.37)	1 (0.36)	
Unknown	27 (10.06)	26 (9.39)	

^*^chi-square tests were used for all comparison of the variables in the table

There was a significant difference between Mwanza and Mbeya (*P* < 0.001) when comparing the number of biopsies performed. Over the four-year span, Mwanza had an average of 225.8 biopsies/year while Mbeya only had an average of 58 biopsies/year.

Table 4, shows no significant difference for Mwanza and Mbeya with respect to age and year of diagnosis for patients who were managed at ORCI compared to patients who were not managed at ORCI. Histopathologic types were significantly different between those who were managed at ORCI compared to those who were managed locally in Mwanza and Mbeya. The statistical significance for histopathologic types is likely a result of a larger proportion of patients with unknown histopathologic data among those who were seen at ORCI compared to the unknown proportion of the patients who were seen locally at Mwanza and Mbeya.

**Table 4:** Differences between Patients who Were Managed at Ocean Road Cancer Institute at those who were Managed Locally in Mwanza and Mbeya, Tanzania

	Managed at ORCI	Managed locally	P^*^
Mwanza			
Age (years)	50.51 ± 12.41	52.50 ± 13.99	0.11
Mbeya			
Age	52.29 ± 11.30	51.12 ± 13.60	0.13
Year of Diagnosis			
Mwanza ^(169,488)^			
	No. (%)	No. (%)	*P*
2014	44 (26.04)	123 (25.20)	0.12
2013	36 (21.30)	147 (30.12)	
2012	45 (26.62)	121 (24.80)	
2011	44 (26.04)	97 (19.88)	
Mbeya^(277,521)^			
2014	66 (23.82)	154 (29.55)	0.09
2013	67 (24.19)	135 (25.91)	
2012	81 (29.25)	113 (21.70)	
2011	63 (22.74)	119 (22.84)	
Hisopthologic Diagnosis^**^			
Mwanza ^(169,488)^			
	No. (%)	No. (%)	*P*
SCC	132 (78.10)	433 (88.70)	< 0.001
AC	14 (8.28)	45 (9.02)	
Others	23 (13.62)	10 (2.00)	
Mbeya ^(277,521)^			
SCC	226 (81.59)	492 (94.40)	< 0.001
AC	21 (7.58)	24 (4.60)	
Others	30 (10.83)	5 (1.00)	

^*^t-test was used for the age comparison and chi-square tests for the remainder of the variables in the table. ^**^ *SCC* Squamous cell carcinoma, *AC* Adenocarcinoma; and Others included unknown, clear cell carcinoma, carcinoma (non-specific), adeno-squamous, and plasmacytoid dendritic cell carcinoma

Table 5, shows the observed and expected incidence rates of cervical cancer for Mwanza and Mbeya. The observed cervical cancer incidence in the regions of Mwanza and Mbeya were underestimated by about 53.9 and 68.9%, respectively.

**Table 5:** Observed Expected Incidence Rates per 100,000 for Mwanza and Mbeya Based on Referred Cases from the Two Regions

Region										
	Mwanza					Mbeya				
Year	2011	2012	2013	2014	P*	2011	2012	2013	2014	P*
Observed incidence	6.97	6.79	6.20	6.20	0.0202	9.84	12.24	9.82	9.38	0.0209
Expected incidence	14.68	14.71	12.95	14.51		34.37	34.44	30.30	33.95	
% Under-estimation	52.50	53.85	52.13	57.2		71.37	64.46	67.61	72.38	

*P values resulted from the Kruskal-Wallis non-parametric test for comparing the observed and expected data

Points between observed and expected incidence for each of the 2 sites

## Discussion

This study revealed three interesting observations. First, based on the ORCI data for the patients treated from the two regions, there were significant underestimations of the incidence rates of cervical cancer in both Mwanza and Mbeya. Second, the majority of the women from Mwanza and Mbeya did not make it ORCI to receive treatment. Third, of the Mwanza and Mbeya cervical cancer patients who received treatment at ORCI, there were no significant differences in the demographic and clinical characteristics of patients between the two regions.

In regards to the first observation of underestimation of incidence rates, both Mwanza and Mbeya had significant underestimation of observed cervical cancer incidence rates based on the data collected from ORCI. We recognized that the majority of the cervical cancer patients who were histopatholgoically-diagnosed in both regions did not seek medical care at ORCI, thus resulting in under-reporting of cervical cancer, based on treatment data from the radiotherapy center (ORCI). This is important because most of Tanzania’s cancer statistics come from ORCI data. Using incidence rates computed from ORCI data creates an inaccurate picture of the true regional incidence for cervical cancer, and possibly other cancers. Lacking a national cancer registry, linkage between pathology laboratories and treatment facilities, like ORCI, is the only way to get clear and accurate incidence rates. This is also essential for future development of population-based cancer registries in Tanzania.

Regarding the second observation that the majority of the women from Mwanza and Mbeya never made it to ORCI to receive treatment. Nearly three-fourths of the women from Mwanza and two-thirds of the women from Mbeya with confirmed histopathologic cervical cancer diagnoses, respectively, never completed their referral to ORCI for treatment. This could be due to a number of factors. First, while the cost of treatment at ORCI is free, for many women in the remote regions of Mwanza and Mbeya, it is too costly in time and money for them to travel the long distance to Dar es Salaam to receive treatment. The distance from Bugando hospital in Mwanza to Ocean Road is 1115 km. Driving, either by car or taking a bus, would take roughly 18 h. Taking the train would take roughly 2 days and cost around 16,900 TSH (~$8). Again, flying is the fastest way to travel, taking only an hour and a half, but costs 150,000 TSH (~$70). The distance from the Mbeya hospital to ORCI is 835 km. Driving, either by car or taking a bus, would take roughly 16 h. Taking train would take roughly a day and a half and cost around 33,000 TSH (~$16.50). Flying is the quickest, taking only an hour and a half, but costing150,000 TSH (~$70). Second, women from those regions could choose not to receive treatment due to the cancer stigma. Third, it is possible that patients do not understand the importance of having the radiotherapy treatment at ORCI or the message about the follow-up treatment at ORCI was not clearly communicated to them after the histopathologic diagnosis in their local hospitals.

It is important to note that Tanzania is observing a steady economic growth. Tanzania’s growth domestic product (GDP) per capita increased from $628 in 2012 to $1105 in 2019. However, the current GDP per capita is still placing Tanzania in the low-income range of countries. The 2019 GDP in million USD PPP of Dar es Salaam in 2019 was 29,585 compared to 16,683 for Mwanza, and 9881 for Mbeya [11–13].

In regards to the third observation, there were no significant differences in the demographic and clinical characteristics of the women who came from Mwanza and Mbeya and received treatment at ORCI. Both Mwanza and Mbeya had the majority of the women who attended ORCI presenting at late stages of the disease, both had the majority of the women receiving a combination of both radiotherapy and chemotherapy, and both regions had the majority of the women with histopathologic diagnosis of squamous cell carcinoma. This was interesting because even though these two regions were not significantly different from one another demographically or clinically, Mbeya’s HIV rate is twice as high as Mwanza’s HIV rate. HIV status was not reported well in medical records at ORCI. Over three-fourths of women from Mwanza and Mbeya had missing HIV status in their ORCI medical records. This made it difficult to get a clear picture of how HIV affects cervical cancer for those patients in the two regions. A previous study investigated the difference in the characteristics of HIV-positive and HIV- negative patients who were diagnosed with cervical cancer in Dar es Salaam, Tanzania [14]. The study found that patients screened for cervical cancer at one of the screening clinics before receiving treatment at ORCI were less likely to have higher disease stages, were more likely to be HIV-positive, and less likely to reside outside of Dar es Salaam [14].

Mwanza had a higher percentage of biopsies that were non-cancerous compared to Mbeya. Based on the data from Bugando hospital in Mwanza where the pathology department examines about 3390 biopsies a year, and for every positive cervical cancer biopsy, there were two negative cervical biopsies. Based on the data from Mbeya hospital, the pathology department averages 1450 biopsies a year, and for every negative cervical biopsy, there were two positive cervical cancer biopsies, the opposite of Mwanza. It is possible that Mwanza hospital screens more women and thus more non-cancerous cases are identified.

The findings from our study agree with the literature on this topic. While no previous studies addressed the research question of this study in Tanzania, a similar study conducted in Zambia investigated the underestimation of cervical cancer incidence in the Southern and Western provinces of Zambia [15]. The study found that cervical cancer was underestimated and that HIV played a significant role [15].

The strengths of this study include the availability of pathology facilities in the study regional hospitals, a large dataset for 4 years, and one treatment radiotherapy center (ORCI) in the entire country during the study period. Other strengths include availability of the referral patterns of patients and detailed information about the patient demographic and clinical information at ORCI and the two distant regional hospitals (Mwanza and Mbeya). The limitations of this study include the portion of missing data in medical records of patients at ORCI and lack of computerized tracking of histopathology records in Mwanza and Mbeya.

It is important to highlight the fact that facilities for radiotherapy treatment in Tanzania, in addition to what is available at ORCI, are just emerging. At Bugando Medical Center in Mwanza, a Cobalt60 machine has just started operation in 2019. No radiotherapy machines exist in Mbeya. Chemotherapy started at the Bugando Medical Center in Mwanza in 2013, in Kilimanjaro Christian Medical Center (KCMC) in 2018, and in Dodoma in 2019. Chemotherapy has also started in a few private hospitals that are not affordable to the low-income Tanzanian population. Small-scale private radiotherapy machines also started on a small scale at two private hospitals in Dar es Salaam in 2017 and 2019. Again, the cost of treatment is beyond the financial affordability of the vast majority of low-income patients in Tanzania who also live outside Dar es Salaam.

## Conclusions

In summary, our study showed a significant underestimation of cervical cancer cases in the two remote regions of Mwanza and Mbeya. There were no significant differences in the demographic and clinical characteristics of the women who originated from Mwanza and Mbeya and received treatment at ORCI. A majority of women who were diagnosed with cervical cancer in both regions did complete their referral to ORCI to receive radiotherapy treatment, and those who received treatment from the two regions at ORCI presented at late stage of the disease. After reviewing the data from ORCI and the referral hospitals, The observed incidence rate in Mwanza and Mbeya were lower than its expected incidence rate for both locations.

Future studies need to explore the possibility of cervical cancer underestimation in other regions of Tanzania, both rural and urban. Other studies need to explore the factors related to non-compliance with referral to ORCI from different regions in Tanzania. Providing local radiotherapy in different regions in Tanzania should be a priority in this large country with limited and costly transportation for the general population. Also, electronic linkage between the different cancer hospitals would definitely improve the quality of future population-based cancer registries in this country.

## Data Availability

The data of this study can be available from Dr. Julius Mwaiselage, Director of the Ocean Road Cancer Institute upon a reasonable request.

## Abbreviations

ORCI: Ocean Road Cancer Institute
MNH: Muhimbili National Hospital
KCMC: Kilimanjaro Christian Medical Center
THMIS: Tanzanian HIV/AIDS and Malaria Indicator Survey
SD: Standard Deviation
